# Correlation between surrogate endpoints and overall survival in unresectable hepatocellular carcinoma patients treated with immune checkpoint inhibitors: a systematic review and meta-analysis

**DOI:** 10.1038/s41598-024-54945-6

**Published:** 2024-02-21

**Authors:** Litao Huang, Deying Kang, Chongyang Zhao, Xueting Liu

**Affiliations:** 1 Chinese Evidence-Based Medicine Center, National Clinical Research Center for Geriatrics, West China Hospital, Sichuan University, Chengdu, Sichuan China; 2 Department of Clinical Research Management, West China Hospital, Sichuan University, Chengdu, 610041 China; 3 Department of Evidence-Based Medicine and Clinical Epidemiology, West China Hospital, Sichuan University, Chengdu, Sichuan China; 4 Discipline Construction Department, West China Hospital, Sichuan University, Chengdu, 610041 China

**Keywords:** Immunology, Hepatocellular carcinoma, Survival, Meta-analysis, Cancer therapy, Tumour immunology, Liver cancer

## Abstract

This study aimed to assess the therapeutic effect of immune checkpoint inhibitors (ICIs) in patients with unresectable hepatocellular carcinoma (uHCC) and investigate the correlation between surrogate endpoints and overall survival (OS). A systematic literature search included phase I, II, and III clinical trials comparing ICIs to placebo or other therapies for uHCC treatment. Correlations between OS and surrogate endpoints were evaluated using meta-regression analyses and calculating the surrogate threshold effect (STE). The correlation analysis showed a weak association between OS and progression-free survival (PFS), with an R^2^ value of 0.352 (95% CI: 0.000–0.967). However, complete response (CR) exhibited a strong correlation with OS (R^2^ = 0.905, 95% CI: 0.728–1.000). Subgroup analyses revealed high correlations between OS and PFS, CR, stable disease (SD), and DC in phase III trials (R^2^: 0.827–0.922). For the ICI + IA group, significant correlations were observed between OS and SD, progressive disease (PD), and grade 3–5 immune-related adverse events (irAEs) (R^2^: 0.713–0.969). Analyses of the correlation between survival benefit and risk of mortality across various time points showed a strong association within the first year (R^2^: 0.724–0.868) but a weak association beyond one year (R^2^: 0.406–0.499). In ICI trials for uHCC, PFS has limited utility as a surrogate endpoint for OS, while CR exhibits a strong correlation with OS. Subgroup analyses highlight high correlations between OS and PFS, SD, and DC in phase III trials. Notably, the ICI + IA group shows significant associations between OS and SD, PD, and grade 3–5 irAEs. These findings offer valuable insights for interpreting trial outcomes and selecting appropriate endpoints in future clinical studies involving ICIs for uHCC patients.

## Introduction

Unresectable hepatocellular carcinoma (uHCC) is generally associated with a poor prognosis^1^. Historically, treatment options for advanced HCC patients, particularly those with metastatic and unresectable disease were limited. However, the landscape of treatment for advanced HCC has significantly transformed in recent years with the introduction of immunotherapy^2,3^. Immunotherapy works by activating the patient's own immune system to fight against tumors. By targeting immune checkpoints, immunotherapy helps to unleash the inhibitory effects that prevent immune cells from effectively recognizing and attacking tumor cells^4,5^. As a result, modern immunotherapy based on immune checkpoint inhibitors (ICIs) has emerged as a promising first-line treatment approach for unresectable HCC, either as monotherapy or in combination with other anticancer agents^6^.

According to both the Food and Drug Administration (FDA) and the European Medicines Agency (EMA), overall survival (OS) is considered remains the gold standard for demonstrating the clinical benefit of new anticancer therapies^7^. However, the interpretability of OS can be influenced by several factors, including prolonged postprogression follow-up time, treatment crossover, and subsequent anticancer therapies^8^. Therefore, surrogate endpoints such as progression-free survival, time to progression, duration of response, and objective response rate are being investigated and used in oncology studies^9,10^. The use of surrogate endpoints offers several advantages over OS^11–13^ . Firstly, it accelerates the research process, expediting the approval and clinical trial processes, resulting in faster completion of clinical trials and reducing the time required for research and development. Secondly, it provides earlier access to new therapies. Through the accelerated process, patients, especially those in urgent need of medical intervention, can gain access to new treatment options at an earlier stage. This allows patients to receive potentially life-saving or life-improving treatments sooner and minimizes any delays in receiving effective therapies. Thirdly, it enhances efficiency and reduces costs. Faster trials enable us to swiftly evaluate clinical pharmacology and mechanisms of action, providing valuable insights for both researchers and physicians. This not only facilitates more efficient screening of potential drug candidates, but also brings cost advantages to pharmaceutical manufacturers or sponsors. Such expedited approval and clinical trial processes allow them to allocate resources more effectively, potentially alleviating the financial burdens associated with drug development.

To date, only 20% of the subsequent clinical trials for 93 cancer drug indications with accelerated FDA approval have shown improved overall survival in patients^14^. Similarly, a review by EMA and NICE found that out of 52 drugs, 43 (82.7%) lacked overall survival data initially, although 9 drugs (17.3%) later demonstrated improved overall survival^15^. In the context of hepatocellular carcinoma (HCC), nivolumab received accelerated approval based on data from the CheckMate 040 trial^16^. This phase I/II clinical study, which was multicenter, prospective, uncontrolled, and open-label, showed that nivolumab treatment in advanced HCC patients was safe, manageable, and improved objective response rates. However, the subsequent clinical trial CheckMate 459, an adaptive-designed, randomized, open-label trial, failed to confirm the survival benefit of nivolumab as a first-line therapy for advanced HCC patients who had not previously received systemic therapy^17^. As a result, the FDA revoked the indication for nivolumab as a first-line therapy for HCC in 2020. In addition, pembrolizumab obtained accelerated approval based on data from the KEYNOTE-224 trial. This phase II prospective, single-arm, open-label study evaluated the safety and efficacy of pembrolizumab in patients with advanced HCC who had previously been treated with sorafenib^18^. Currently, the approval status of pembrolizumab remains valid without being withdrawn. However, second-line pembrolizumab showed a large difference in improving overall survival (OS) between Asian and non-Asian populations^19,20^. Furthermore, multiple ongoing trials aim to deepen our understanding of the molecular pathogenesis of uHCC and explore new therapeutic approaches for this condition^21^. As a result, the selection of alternative indicators for uHCC becomes particularly important.

The aim of this study was to assess whether the therapeutic effect on surrogate endpoints at the trial level can predict the therapeutic effect on OS. A secondary objective was to explore heterogeneity in trial-level correlations based on specific trial characteristics. To this end, we conducted a systematic literature search and trial-based meta-analysis of experimental ICI therapy in patients with uHCC.

## Materials and methods

The Preferred Reporting Items for Systematic Reviews and Meta-Analyses (PRISMA) statement was used for this systematic review^22^.The meta-analysis protocol was submitted to PROSPERO: CRD42023433976.

### Search for studies

In our search for relevant trials, we will utilize a comprehensive approach. We will explore multiple databases, including the Cochrane Central Register of Controlled Trials (CENTRAL, Ovid), MEDLINE (PubMed), and EMBASE (Ovid) (refer to eTable 1 for the search strategy). Furthermore, we will conduct searches on online trial registries such as ClinicalTrials.gov, European Medicines Agency (EMA), and WHO International Clinical Trial Registry Platform. Additionally, in our quest for valuable information, we will search for grey literature in abstracts and posters presented at the American Society of Clinical Oncology (ASCO) annual congress via the System for Information.

It is important to note that this search strategy was performed on 9 June 2023.

### Types of study to be included

For the inclusion criteria of our study, we will consider all phase I, II, and III clinical trials, as well as expanded access programs (external clinical trials). These trials and programs should involve a comparison between immune checkpoint inhibitors and placebo, no treatment, or systemic/locoregional therapies. The focus of our study is the treatment of uHCC.

### Data extraction

The literature search was conducted using the literature management software Endnote X9. After removing duplicates, two researchers (LTH and CYZ) independently screened the search results in two rounds. In the first round, titles and abstracts were reviewed to exclude irrelevant literature or literature that did not meet the inclusion criteria. The full texts of the remaining studies were then read in the second round to complete the selection. In case of disagreement between the two researchers, a third and more senior researcher (XTL) was consulted to make a final judgement. Excluded studies and reasons for exclusion will be documented.

We will extract the data individually. We will extract details of the study population, interventions, and outcomes using a piloted, standardized data extraction form. This form will include the following items:

1. Study methods and characteristics: such as study design, country, target disease, year of publication, funding type, study registration number, inclusion and exclusion criteria, sample size, and population characteristics;
2. Severity of illness: Child–Pugh score, Eastern Cooperative Oncology Group Performance Status, Barcelona Clinic Liver Cancer stage, proportion of participants’ positive for hepatitis B and hepatitis C virus;
3. Experimental and control arms of the trial;
4. Outcomes:
  1. Overall survival (OS), defined as the time from randomization until death from any cause, commonly used in phase III trials.
  2. Time to progression of the tumor: progression-free survival (PFS), time from randomization to objective tumor progression or death.
  3. Tumor response assessments (as recommended by the response evaluation in solid tumors criteria (RECIST1.1)^23^).
    Proportion of people with objective response (OR), complete response (CR), partial response (PR), progressive disease (PD), stable disease (SD), and disease control (DC).
  4. Adverse events: Proportion of participants with one or more nonserious immune-related adverse events (irAEs) based on the National Cancer Institute Common Terminology Criteria for Adverse Events (CTCAE) and/or the Medical Dictionary for Regulatory Activities.

### Statistical analysis

We firstly conducted a narrative description of the included studies. Categorical variables are reported as frequencies and percentages, and continuous variables are reported as medians with interquartile ranges (IQRs) unless indicated otherwise.

In each trial, we abstracted the hazard ratios (HRs) along with their corresponding 95% confidence intervals (CIs) and survival rates at specific time points^24^. In cases where HRs were not explicitly provided, we estimated them based on relevant effect measures derived from the given median survival times or survival rates at those time points. A frequentist hybrid model for random effect multivariate meta-analysis was used to evaluate surrogacy between the HRs for OS and each endpoint. A Bayesian hybrid model random-effects multivariate meta-analysis was used for sensitivity analysis. No covariates were used in the hybrid models for random effect multivariate meta-analysis. A random effects meta-regression model was used to quantify the association between the natural logarithm of the HRs for OS and each endpoint (The data analysis flowchart is shown in eFig. 1). According to the ReSEEM (Systematic Review and Recommendation for Reporting of Surrogate Endpoint Evaluation using Meta-analyses) guidelines^25^, R^2^ values ≥ 0.7 represent strong correlations (and thus suggest surrogacy), values between 0.69 and 0.5 represent moderate correlations, and values < 0.5 represent weak correlations. After implementing the meta-regression model, our next step is to calculate the surrogate threshold effect (STE), which plays a crucial role in determining the thresholds for estimating the surrogate endpoint^26^. In addition, we planned to investigate the impact of clinical information on the association between overall survival (OS) and surrogate measures through subgroup analyses of predefined subgroups. All statistical analyses were performed using R ver. 4.3.0 software.

## Results

### Characteristics and quality assessment

**Figure 1:**
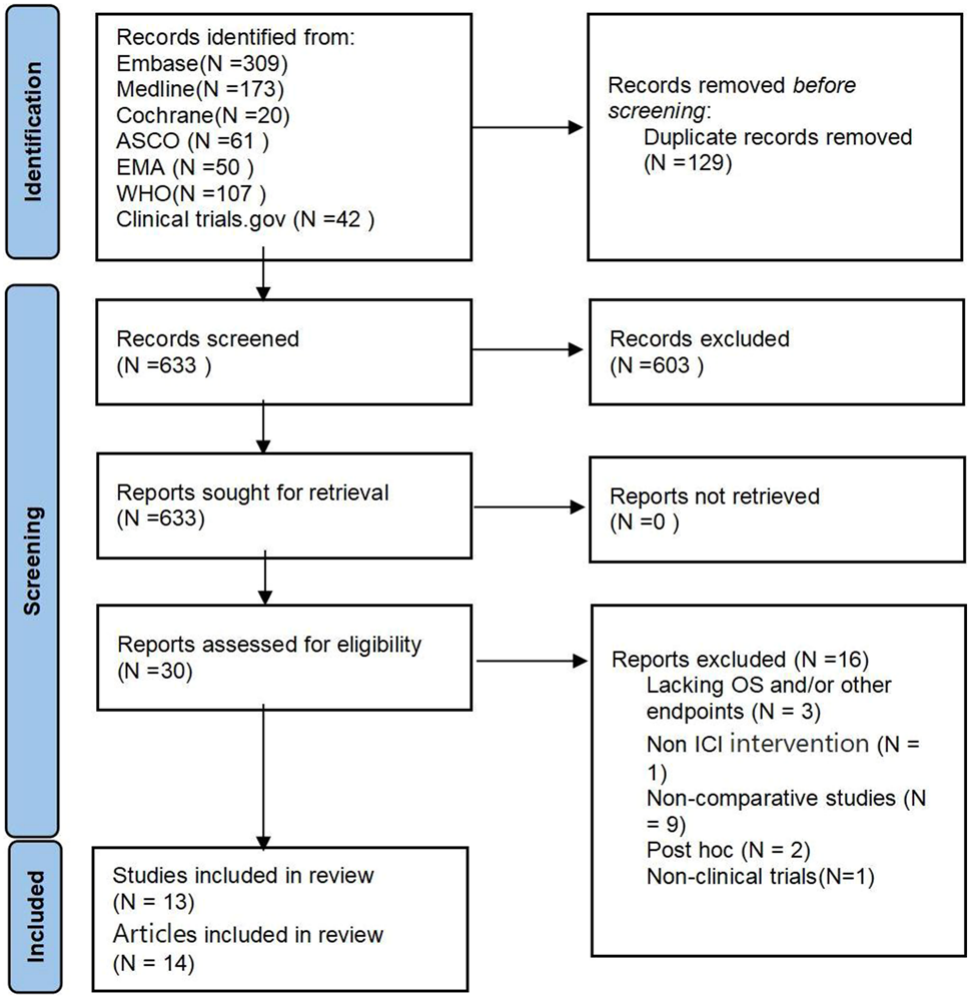
Flow diagram of study selection process. *N* number of patients.

The systematic review process, as depicted in Fig. 1, enabled us to identify a total of 13 studies from the 14 articles reviewed. The inclusion and exclusion of full-text articles are listed in eTable 2. Among the 13 reported trials, 9 were obtained from full-text reports, for which we conducted a risk of bias assessment. It was found that the overall risk of bias was low, with the main concern being open-label studies (eFig. 2). Notably, the study IMbrave15_update26^27^ provided additional data during an extended follow-up period, leading to its inclusion in our analysis. Across these 13 studies, we included a total of 20 comparison subgroups, involving a cohort of 4573 patients, as detailed in Table 1.

**Table 1:** Randomised clinical trials characteristics.

Name	Year	Source	Phase	Sample size	Randomization ratio	Intervention	Line of therapy	Blinding	Primary end points	ECOG = 0 (%)	Child Pugh classification (%)	Barcelona Clinic liver cancer stage (%)	HBV (%)	HCV (%)
IMbrave150^43^	2020	Full text	3	326 vs 159	2: 1	ICI + AI	1	Open label	OS、PFS	62	A:100	A:2; B:16; C:82	47.9	21.6
IMbrave15_update^27^	2022	Full text	3	326 vs 159	2: 1	ICI + AI	1	Open label	OS、PFS	62	A:101	A:2; B:16; C:82	47.9	21.6
GO30140_group F^44^	2020	Full text	1	60 vs 59	1: 1	ICI + AI	1	Open label	PFS	38.7	A:100	A:1.7; B:8.4; C:90	55.5	17.6
Qin (2020)^45^	2020	Full text	2	109 vs 108	1: 1	ICI	2 +	Open label	ORR, OS	21	A:98	B:5; C:95	83	
COSMIC-312 (1)^46^	2020	Full text	2	432 vs 217	2: 1	ICI + TKI	1	Open label	PFS、OS	64.9	A:100	B:32.7; C:64.3	29	31
COSMIC-312 (2)^46^	2020	Full text	2	432 vs 188	2: 1	ICI + TKI	1	Open label	PFS、OS	65	A:100	B:33.2; C:66.8	30	31.6
CheckMate 040 (1)^16^	2020	Full text	1/2	50 vs 49	1: 1	ICI + ICI	1 +	Open label	Safety and tolerability、ORR		A:98	A:2; B:8.1; C:88.9	49.5	21.2
CheckMate 040 (2)^16^	2020	Full text	1/2	50 vs 49	1: 1	ICI + ICI	1 +	Open label	Safety and tolerability、ORR		A:98	A:2; B:7; C:90	54.5	19.2
KEYNOTE-240^19^	2020	Full text	3	278 vs 135	2: 1	ICI + BSC	2	Double blind	OS、PFS	56.4	A:99.3	B:20.6; C:79.4	24.5	15.5
CheckMate 459^17^	2022	Full text	3	371 vs 372	1: 1	ICI	1	Open label	OS	71.6	A:97.2	A:3.9; B:15.6; C:79.9	46.1	23.3
Qin (2023)^20^	2023	Full text	3	300 vs 153	2: 1	ICI + BSC	2	Double blind	OS	40.6	A:100	B:6.6; C:93.4	79.5	1.3
ORIENT-32^47^	2021	Full text	2/3	380 vs 191	2: 1	ICI + AI	1	Open label	OS、PFS	50	A:95.8; B:4.2	B:14.5; C:85.5	94	2.4
HIMALAYA (1)^48^	2022	Abstract	3	393 vs 389	1: 1	ICI + ICI	1	Open label	OS	83.1				
HIMALAYA (2)^48^	2022	Abstract	3	389 vs 389	1: 1	ICI	1	Open label	OS					
NCT02859324 (1)^49^	2020	Abstract	1/2	7 vs 9	1: 1	ICI + IMiDs	Unclear	Open label	DLTS					
NCT02859324 (2)^49^	2020	Abstract	1/2	7 vs 5	1: 1	ICI + IMiDs	Unclear	Open label	DLTS					
EUCTR2015-005417–76 (1)^50^	2021	Abstract	1/2	6 vs 10	1: 1	ICI + TKI	Unclear	Open label	DLTS					
EUCTR2015-005417-76 (2)^50^	2021	Abstract	1/2	6 vs 11	1: 1	ICI + TKI	Unclear	Open label	DLTS					
EUCTR2015-005417-76 (3)^50^	2021	Abstract	1/2	32 vs 30	1: 1	ICI + TKI	Unclear	Open label	DLTS					
NCT02837029^51^	2020	Abstract	1	5 vs 5	1: 1	ICI	Unclear	Open label	DLTS					

*OS* overall survival, *PFS* progression free survival, *ORR* objective response rate, *DLT* dose-limiting toxicity, *ICI* immune checkpoint inhibitor, *AI* angiogenesis inhibitor, *TKI* tyrosine kinase inhibitor, *IMiD* immunomodulatory drugs, *BSC* best supportive care treatment.

Among the 13 trials included in the analysis, five (38.5%) were phase 3 clinical trials conducted to confirm the efficacy of the treatments. It is interesting to note that in three of these trials (23.1%), overall survival (OS) was defined as the only primary endpoint. Additionally, four of the included studies (30.8%) considered OS as a dual/coprimary endpoint alongside progression-free survival (PFS). Table 1 provides a clear overview of the primary endpoints used in these trials, with PFS being the most commonly used.

Upon examining the characteristics of the patients included in the studies, it was found that the majority of them had Eastern Cooperative Oncology Group performance status classified as 0, Child–Pugh scores categorized as A, and Barcelona Clinic Liver Cancer stage B–C. However, it is crucial to acknowledge the heterogeneity in the etiology of hepatocellular carcinoma (HCC) among these studies. The percentage of patients with hepatitis B virus (HBV) ranged from 24.5 to 94%, while those with hepatitis C virus (HCV) ranged from 1.3 to 31.6%.

### Main analysis

Table 2 presents the correlations between OS and potential surrogate outcomes in trials of ICIs in patients with uHCC.

**Table 2:** Relationship between overall survival (OS) and potential surrogate outcomes.

	Number of comparisons analysed	Number of patients		Slope	Formula	R^2^	STE	R
PFS	9	4135	Frequency	0.524 (−0.672, 1.719)	Y = −0.086 + 0.524X	0.352 (0.000, 0.967)	0.165	0.593 (0.000, 0.902)
			Bayes	0.562 (−0.264, 1.285)	Y = −0.082 + 0.562X	0.208 (0.001, 0.514)	0.146	
OR	9	4135	Frequency	−0.117 (−0.421, 0.187)	Y = −0.155–0.117X	0.106 (0.000, 0.571)	−1.322	0.325 (0.000, 0.814)
			Bayes	−0.116 (−0.335, 0.0977)	Y = −0.158–0.116X	0.235 (0.001, 0.541)	−1.353	
CR	7	3347	Frequency	−0.131 (−0.351, 0.090)	Y = −0.079–0.131X	0.905 (0.728, 1.000)	−0.528	0.951 (0.699, 0.993)
			Bayes	−0.134 (−0.233, −0.033)	Y = −0.071–0.134X	0.834 (0.391, 0.900)	−0.606	
PR	9	4135	Frequency	−0.0645 (−0.328, 0.199)	Y = −0.208–0.065X	0.030 (0.000, 0.297)	−3.225	0.172 (0.000, 0.750)
			Bayes	−0.0641 (−0.273, 0.141)	Y = −0.210–0.064X	0.163 (0.000, 0.473)	−3.274	
SD	9	4135	Frequency	0.239 (−0.718, 1.197)	Y = −0.274 + 0.239X	0.007 (0.000, 0.139)	1.145	0.083 (0.000, 0.708)
			Bayes	0.250 (−0.465, 1.046)	Y = −0.276 + 0.250X	0.197 (0.000, 0.504)	1.106	
DC	9	4135	Frequency	−0.548 (−2.452, 1.356)	Y = −0.184–0.548X	0.225 (0.000, 0.814)	−0.336	0.475 (0.000, 0.866)
			Bayes	−0.571 (−2.025, 1.007)	Y = 0.184–0.571X	0.141 (0.000, 0.436)	−0.323	
PD	9	4135	Frequency	0.0255 (−1.104, 1.155)	Y = −0.270 + 0.026X	0.225 (0.000, 0.814)	10.580	0.475 (0, 000, 0.866)
			Bayes	0.0162 (−1.042, 0.936)	Y = −0.273 + 0.016X	0.117 (0.000, 0.391)	16.854	
Any grade irAEs	7	3799	Frequency	−0.164 (−1.648, 1.320)	Y = −0.326–0.164X	0.026 (0.000, 0.331)	−1.921	0.160 (0.000, 0.815)
			Bayes	−0.171 (−1.501, 1.294)	Y = −0.328–0.171X	0.148 (0.000, 0.435)	−2.056	
Grade 3–4 irAEs	8	3918	Frequency	−0.004 (−0.334, 0.326)	Y = −0.300–0.004X	0.026 (0.000, 0.303)	−76.846	0.162 (0.000, 0.778)
			Bayes	−0.004 (−0.262, 0.299)	Y = −0.302–0.004X	0.135 (0.000, 0.422)	−68.992	
Grade 5 irAEs	5	2933	Frequency	0.599 (−1.234, 2.432)	Y = −0.355 + 0.599X	0.351 (0.000, 1.000)	0.593	0.592 (0.000, 0.9685)
			Bayes	0.590 (−2.106, 3.420)	Y = −0.357 + 0.590X	0.331 (0.002, 0.599)	0.606	

*PFS* progression-free survival, *OR* objective response, *CR* complete response, *PR* partial response, *SD* stable disease, *DC* disease control, *PD* progressive disease, *irAE* immune-related adverse event.

We conducted a meta-regression analysis to examine the association between the natural logarithm of HR for OS and PFS, adjusting for estimation errors. The resulting equation was: (log HR of OS) = −0.086 + 0.524 (log HR of PFS), indicating a predicted 52.4% increase in the log HR of OS for each unit increase in the log HR of PFS. To further evaluate the relationship, we calculated the surrogate threshold effect (STE), which was found to be 0.165. Moreover, we observed a notably weak association between PFS and OS. This was reflected in an R^2^ value of 0.352 (95% CI: 0.000–0.967), indicating that only 35.2% of the variability in the effects on OS could be attributed to the observed effects on PFS.

In addition to examining the relationship between OS and PFS, we also explored how treatment effects on OS relate to various tumor response assessment endpoints, including OR, CR, PR, SD, DC, and PD. Our analysis revealed a generally weak association between treatment effects on OS and the majority of these response endpoints, with R^2^ values ranging from 0.007 to 0.225. However, in regard to CR, we observed a strong correlation between treatment effects on CR and OS. The association between the logarithm of relative risk for CR and the logarithm of hazard ratio for OS yielded an R^2^ value of 0.905 (95% CI: 0.728–1.000). In the regression model, the equation for the association between OS and CR was (log HR of OS) = −0.079 to 0.131 (log RR of CR). The analysis also included a regression of log HR for OS on log RR for irAEs, which showed consistently weak correlations with R^2^ values ranging from 0.026 to 0.351, indicating that less than one-third of the variability in survival benefit from ICI therapy can be explained by the variability in treatment effects on irAEs.

Furthermore, the sensitivity Bayesian analysis, conducted across all of the aforementioned analyses, also yielded similar results.

### Subgroup and sensitivity analyses

Table 3 presents the results of our examination of the correlation between OS and PFS, OR, CR, PR, SD, DC, PD, or irAEs by stratum. We found a strong correlation between OS and PFS in phase III trials (R^2^: 0.851, 95% CI: 0.469–1.000). Additionally, we observed high estimated correlations between OS and SD as well as CD in phase III trials. The R^2^ for OS and SD was found to be 0.890 (95% CI: 0.602–1.000), indicating a strong correlation. Similarly, the R^2^ for OS and CD was 0.827 (95% CI: 0.391–1.000), demonstrating a significant association between these variables.

**Table 3:** Overview of sensitivity analyses.

		PFS	OR	CR	PR	SD	DC	PD	Any grade irAEs	Grade 3–4 irAEs	Grade 5 irAEs
Sensitivity analysis 1											
Sampel size > 315^a^	Slope (95% CI)	0.748 (−0.714, 2.210)	−0.091 (−0.415, 0.234)	−0.169 (−0.501, 0.162)	0.047 (−0.317, 0.223)	0.010 (−0.991, 1.190)	−0.910 (−2.949, 1.132)	0.203 (−1.014, 1.419)	−0.164 (−1.648, 1.320)	−0.121 (−0.529, 0.286)	0.599 (−1.234, 2.432)
	R^2^	0.429 (0.000, 1.000)	0.075 (0.000, 0.518)	0.935 (0.791, 1.000)	0.019 (0.000, 0.256)	0.000 (0.000, 0.024)	0.319 (0.000, 0.993)	0.027 (0.000, 0.309)	0.026 (0.000, 0.331)	0.093 (0.000, 0.634)	0.351 (0.000, 1.000)
	R	0.655 (0.000, 9.430)	0.273 (0.000, 0.820)	0.967 (0.720, 0.997)	0.138 (0.000, 0.768)	0.013 (0.000, 0.711)	0.565 (0.000, 0.908)	0.165 (0.000, 0.779)	0.16 (0.000, 0.815)	0.305 (0.000, 0.860)	0.592 (0.000, 0.969)
	Number of comparisons analysed	7	8	6	8	8	8	8	7.00	7.00	5.00
	Number of patients	3799	4016	3228	4016	4016	4016	4016	3799.00	3799.00	2933.00
Sensitivity analysis 2											
Phase:2+	Slope (95% CI)	0.843 (−0.642, 2.327)	0.011 (−0.477, 0.500)	−0.178 (−0.566, 0.209)	0.030 (−0.313, 0.372)	−0.969 (−2.943, 1.006)	−0.990 (−3.041, 1.061)	0.485 (−0.845, 1.815)	−0.209 (−1.700, 1.283)	−0.175 (−0.605, 0.255)	0.4649 (−1.449, 2.378)
	R^2^	0.899 (0.683, 1.000)	0.0008 (0.000, 0.064)	0.922 (0.715, 1.000)	0.008 (0.000, 0.205)	0.676 (0.073, 1.000)	0.659 (0.033, 1.000)	0.412 (0.000, 1.000)	0.092 (0.000, 0.7158)	0.495 (0.000, 1.000)	0.229 (0.000, 1.000)
	R	0.948 (0.594, 0.995)	0.028 (0.000, 0.821)	0.960 (0.510, 0.998)	0.088 (0.000, 0.840)	0.822 (0.032, 0.980)	0.812 (0.001, 0.979)	0.642 (0.000, 0.956)	0.304 (0.000, 0.895)	0.704 (0.000, 0.964)	0.479 (0.000, 0.986)
	Number of comparisons analysed	6	6	5	6	6	6	6	6	6	4
	Number of patients	3150	3150	2579	3150	3150	3150	3150	3150	3150	2284
Phase:3	Slope (95% CI)	0.792 (−1.003, 2.586)	0.073 (−0.441, 0.588)	−0.178 (−0.566, 0.209)	0.061 (−0.290, 0.413)	−0.900 (−2.887, 1.088)	−0.903 (−2.975, 1.169)	0.439 (−0.898, 1.777)	−1.72 (−1.668, 1.323)	−0.136 (−0.600, 0.327)	0.254 (−1.946, 2.454)
	R^2^	0.851 (0.469, 1.000)	0.066 (0.000, 0.728)	0.922 (0.715, 1.000)	0.110 (0.000, 0.928)	0.890 (0.602, 1.000)	0.827 (0.391, 1.000)	0.503 (0.000, 1.000)	0.090 (0.000, 0.849)	0.4 (0.000, 1.000)	0.08
	R	0.922 (0.215, 0.995)	0.256 (0.000, 0.929)	0.96 (0.510, 0.998)	0.332 (0.000, 0.940)	0.943 (0.364, 0.996)	0.909 (0.137, 0.994)	0.709 (0.000, 0.979)	0.301 (0.000, 0.935)	0.632 (0.000, 0.972)	0.28
	Number of comparisons analysed	5	5	5	5	5	5	5	5	5	3
	Number of patients	2579	2579	2579	2579	2579	2579	2579	2579	2579	1713
Sensitivity analysis 3											
Double blind	Slope (95% CI)	0.379 (−30.541, 31.300)	0.013 (−1.080, 1.107)	−0.016 (−1.298, 1.266)	0.008 (−0.615, 0.630)	−0.166 (−13.705, 13.372)	−0.155 (−12.791, 12.481)	0.065 (−5.255, 5.386)	0.052 (−4.235, 4.340)	−0.007 (−0.608, 0.593)	NA
	R^2^	NA	NA	NA	NA	NA	NA	NA	NA	NA	NA
	R	1 (NaN, NaN)	1 (NaN, NaN)	1 (NaN, NaN)	1 (NaN, NaN)	1 (NaN, NaN)	1 (NaN, NaN)	1 (NaN, NaN)	1 (NaN, NaN)	1 (NaN, NaN)	NA
	Number of comparisons analysed	2	2	2	2	2	2	2	2	2	0
	Number of patients	866	866	866	866	866	866	866	866	866	0
Open label	Slope (95% CI)	0.520 (−0.686, 1.726)	−0.253 (−0.671, 0.165)	−0.135 (−0.359, 0.089)	−0.222 (−0.662, 0.217)	0.302 (−0.718, 1.322)	−0.551 (−2.528, 1.427)	0.028 (−1.129, 1.185)	−0.794 (−2.938, 1.351)	0.041 (−0.430, 0.511)	0.599 (−1.234, 2.432)
	R^2^	0.342 (0.000, 1.000)	0.338 (0.000, 1.000)	0.938 (0.772, 1.000)	0.209 (0.000, 0.917)	0.016 (0.000, 0.263)	0.216 (0.000, 0.930)	0.012 (0.000, 0.225)	0.162 (0.000, 1.000)	0.012 (0.000, 0.252)	0.351 (0.000, 1.000)
	R	0.585 (0.000, 0.929)	0.581 (0.000, 0.928)	0.969 (0.594, 0.998)	0.457 (0.000, 0.900)	0.128 (0.000, 0.804)	0.465 (0.000, 0.902)	0.110 (0.000, 0.797)	0.402 (0.000, 0.948)	0.108 (0.000, 0.845)	0.592 (0.000, 0.969)
	Number of comparisons analysed	7	7	5	7	7	7	7	5	6	5
	Number of patients	3269	3269	2481	3269	3269	3269	3269	2933	3052	2933
Sensitivity analysis 4											
Line of therapy:1	Slope (95% CI)	0.414 (−0.982, 1.809)	−0.332 (−0.968, 0.305)	−0.135 (−0.359, 0.089)	−0.248 (−0.915, 0.42)	0.131 (−1.178, 1.44)	−0.508 (−2.49, 1.474)	0.088 (−1.081, 1.257)	−0.793 (−2.938, 1.351)	0.041 (−0.43, 0.511)	0.599 (−1.234, 2.432)
	R^2^	0.282 (0, 1)	0.286 (0, 1)	0.938 (0.772, 1)	0.121 (0, 0.814)	0.004 (0, 0.149)	0.234 (0, 1)	0.023 (0, 0.359)	0.162 (0, 1)	0.012 (0, 0.252)	0.351 (0, 1)
	R	0.531 (0, 0.938)	0.534 (0, 0.939)	0.969 (0.594, 0.998)	0.348 (0, 0.904)	0.064 (0, 0.832)	0.484 (0, 0.93)	0.152 (0, 0.858)	0.402 (0, 0.948)	0.108 (0, 0.845)	0.592 (0, 0.968)
	Number of comparisons analysed	6	6	5	6	6	6	6	5	6	5
	Number of patients	3052	3052	2481	3052	3052	3052	3052	2933	3052	2933
Line of therapy:2	Slope (95% CI)	0.38 (−30.541, 31.3)	0.013 (−1.08, 1.107)	−0.016 (−1.298, 1.266)	0.008 (−0.615, 0.63)	−0.166 (−13.705, 13.372)	−0.155 (−12.791, 12.481)	0.065 (−5.255, 5.386)	0.053 (−4.235, 4.34)	−0.007 (−0.608, 0.593)	NA
	R^2^	1 (1, 1)	1 (1, 1)	1 (1, 1)	1 (1, 1)	1 (1, 1)	1 (1, 1)	1 (1, 1)	1 (1, 1)	1 (1, 1)	NA
	R	1 (NaN, NaN)	1 (NaN, NaN)	1 (NaN, NaN)	1 (NaN, NaN)	1 (NaN, NaN)	1 (NaN, NaN)	1 (NaN, NaN)	1 (NaN, NaN)	1 (NaN, NaN)	NA
	Number of comparisons analysed	2	2	2	2	2	2	2	2	2	0
	Number of patients	866	866	866	866	866	866	866	866	866	0
Sensitivity analysis 5											
Intervention: ICI + AI	Slope (95% CI)	−0.765 (−5.94, 4.409)	−0.321 (−0.973, 0.33)	−0.125 (−0.362, 0.113)	−0.241 (−0.929, 0.447)	1.214 (−0.957, 3.384)	2.303 (−4.571, 9.176)	−2.203 (−6.072, 1.665)	−4.408 (−40.082, 31.266)	0.455 (−0.345, 1.256)	0.333 (−1.601, 2.267)
	R^2^	0.01 (0, 0.397)	0.485 (0, 1)	0.944 (0, 1)	0.249 (0, 1)	0.713 (0, 1)	0.277 (0, 1)	0.816 (0.166, 1)	0.487 (0, 1)	0.766 (0, 1)	0.969 (0, 1)
	R	0.1 (0, 0.968)	0.696 (0, 0.993)	0.972 (0, NaN)	0.499 (0, 0.987)	0.844 (0, 0.997)	0.526 (0, 0.988)	0.904 (0, 0.998)	0.698 (0, NaN)	0.875 (0, 0.997)	0.985 (0, NaN)
	Number of comparisons analysed	4	4	3	4	4	4	4	3	4	3
	Number of patients	1660	1660	1089	1660	1660	1660	1660	1541	1660	1541
Intervention:ICI	Slope (95% CI)	−3.069 (−30.296, 24.157)	−0.086 (−0.844, 0.673)	NA	−0.091 (−0.897, 0.715)	0.133 (−1.045, 1.31)	0.473 (−3.726, 4.673)	−0.204 (−2.012, 1.604)	NA	NA	NA
	R^2^	1 (1, 1)	1 (1, 1)	NA	1 (1, 1)	1 (1, 1)	1 (1, 1)	1 (1, 1)	NA	NA	NA
	R	1 (NaN, NaN)	1 (NaN, NaN)	NA	1 (NaN, NaN)	1 (NaN, NaN)	1 (NaN, NaN)	1 (NaN, NaN)	NA	NA	NA
	Number of comparisons analysed	2	2	1	2	2	2	2	1	1	1
	Number of patients	960	960	743	960	960	960	960	743	743	743

*NE* not evaluable, *NAN* not a number, *ICI* immune checkpoint inhibitor, *AI* antiangiogenic agents.

^a^The median sample size of the included comparison arms was 315, and we performed sensitivity analyses 1 only for studies with larger sample sizes.

In the subgroup analyses of different intervention groups, we uncovered significant correlations between OS and SD, PD, grade 3–4 irAEs, or grade 5 irAEs specifically in the ICI + IA group. The R^2^ observed were as follows: 0.713 (95% CI: 0.00–1.00) for OS and SD, 0.816 (95% CI: 0.166–1.00) for OS and PD, 0.766 (95% CI: 0.00–1.00) for OS and grade 3–4 irAEs, and 0.969 (95% CI: 0.00–1.00) for OS and grade 5 irAEs. These results indicate a strong relationship between these endpoints within the ICI + IA group. However, due to the insufficient sample size, we lacked adequate information to analyze the correlations in other intervention groups.

Correlation between survival benefit and risk of mortality or disease progression across time points.

**Figure 2:**
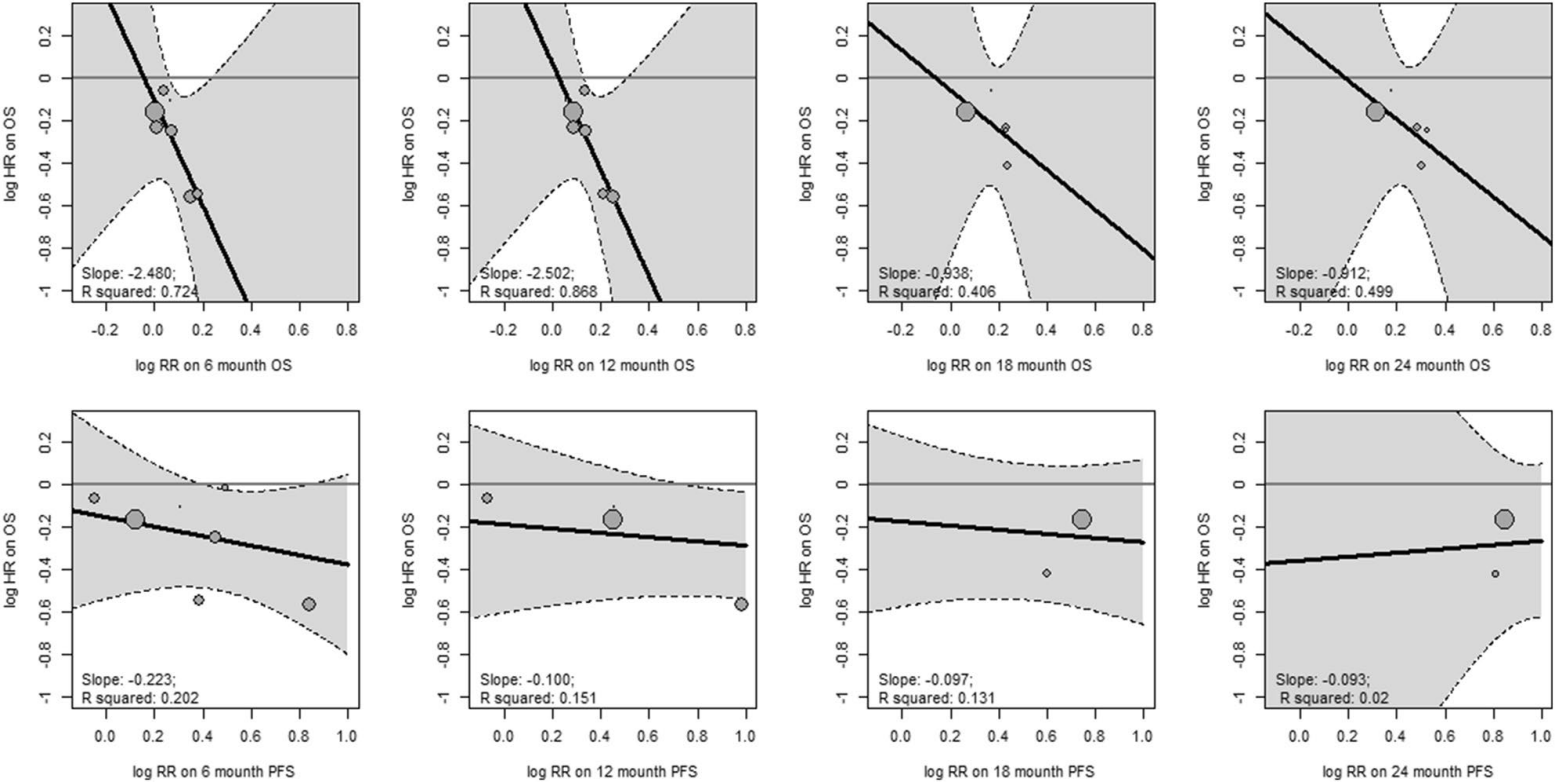
Correlation between survival benefit and risk of mortality or disease progression across time points. Each circle represents a trial, and the surface area of the circle is proportional to the number of events observed in the corresponding trial. Straight lines represent weighted regression lines.

We conducted an analysis to examine the correlation between the survival benefit endpoint (HR for OS) and the relative risk (RR) for both PFS and OS at different time points (6 months, 12 months, 18 months, and 24 months) to investigate the influence of time factors (Fig. 2). We found a strong association between the risk of near-term mortality (within one year) and the survival benefit, with R^2^ values ranging from 0.724 (at 6 months) to 0.868 (at 12 months). However, we observed a weak association between the risk of disease progression and the survival benefit, regardless of whether it was within one year or after one year, with R^2^ values ranging from 0.020 to 0.202.

## Discussion

PFS has historically been the most commonly used alternative endpoint in phase III clinical trials because it is defined as a composite endpoint that combines progression and death^28^. We found that overall the surrogate relationship between the treatment effects on ICIs and the effects on PFS or OS was weak, which supports existing knowledge in this area^29–31^. However, PFS in all these trials was defined using the traditional RECIST criteria, which were developed in the era before immunotherapy. It has been reported that traditional RECIST criteria fail to properly capture the concept of disease progression with immunotherapies that have atypical response patterns^32^. That is failure of traditional RECIST criteria to define PFS of immunotherapies might be a reason for smaller benefits in PFS vs OS with the trials of PD-1 inhibitors. In addition, exploring the relationship between the two through study design found some evidence that the study design may help improve the strength of the PFS-OS surrogacy patterns in uHCC. This could be attributed to the characteristics of phase II clinical trials, which often involve a smaller number of patients, shorter follow-up periods, and shorter trial durations. The influence of random noise may be more pronounced, resulting in a weaker correlation between progression-free survival (PFS) and overall survival (OS) ^33^. On the other hand, phase III clinical trials have larger sample sizes, longer trial durations, and more diverse patient populations. These large-scale trials allow for a more accurate evaluation of treatment effects on PFS and OS, thus typically demonstrating a stronger correlation between the two endpoints.

The number of people achieving ORR is the sum of CR and PR, and the number of people achieving DCR is the sum of CR, PR and SD, which are early-phase outcomes available in most trials^34^. However, the trial-level association results between OS and tumor response end points did not meet the lowest evaluation criteria in the included meta-analyses (R^2^ < 0.60). This phenomenon may be relatedto two reasons: the first is the mechanism of immunotherapy. There is a phenomenon known as pseudoprogression, in which the tumor may show local enlargement or the appearance of new lesions at the beginning of treatment, but then significant therapeutic effects can appear^35,36^. In this case, patients may experience poor disease control for a certain period of time but eventually achieve a good treatment response and extended overall survival. Therefore, the correlation between OS and OR or DC can be affected by pseudoprogression. In fact, low correlations between OS and traditional alternative endpoints are not uncommon in other treatments in chemotherapy^10,37,38^. The second reason may be related to the importance of liver decompensation as a driving factor for the death of HCC patients. Unlike PFS, liver decompensation cannot be directly captured through radiological assessment. Therefore, the potential impact of liver decompensation limits the full understanding of the relationship between radiological endpoints and OS in HCC patients^39^. In contrast, for CR, its definition is clearer and usually requires the patient's disease to disappear for a period of time. Complete reduction indicates that the treatment has a very good control effect on the tumor^8^. Therefore, although achieving complete remission is rare, it does not exclude the possibility of clinical cure for patients who achieve this outcome. This perspective is consistent with the current clinical reality and is not contradictory to existing evidence. Patients may have a higher chance of achieving long-term disease-free survival, signifying that complete remission reflects a more comprehensive treatment response and tumor control and may be directly related to overall survival.

In the treatment of advanced cancer with ICIs, the association between the occurrence of adverse reactions and OS is generally low. ICIs generally have better safety and tolerability than traditional chemotherapy drugs, and the adverse reactions are caused by the overactivity of the immune system caused by the treatment^40^. ICIs enhance the immune response by inhibiting inhibitory signals on the immune system and may trigger irAEs, such as immune cells attacking normal tissues due to excessive activation^41^. In our study, we observed a strong correlation between irAEs and OS specifically in the ICI + AI completion group, suggesting that this association may vary based on the treatment approach. However, due to the wide confidence intervals for the estimated R^2^ values, further validation is needed for all subgroup analyses, and the small sample size may not capture true associations. Therefore, adverse reactions do not directly reflect the effect of treatment on the tumor.

This meta-analysis has several limitations that need to be considered when interpreting the study results. Firstly, the lack of individual patient-level data limits our ability to account for potential confounding factors known to influence OS. Although we intend to investigate this further in future research, the absence of this data is a limitation of the current study. Secondly, not all studies included in the analysis reported all secondary endpoints at the time of analysis, which may introduce publication bias. While efforts were made to estimate HRs from available data, the assumption that HRs can be estimated from median survival times or survival at a specific time point may be overly simplified and may not capture the complete picture. Thirdly, we acknowledge that using HRs as the sole measure to assess the correlation between surrogate endpoints and OS has its limitations. HR is influenced by both the experimental arm and the control arm results, and the efficacy of the control arm can vary across different RCTs. Violation of the assumption of proportional hazards, which is necessary for using HRs, is often observed in RCTs of advanced HCC^42^. However, given that this study was a secondary analysis without access to raw data for adjustment or consideration of all potential confounding factors, we chose to use the available HRs for practical reasons. Furthermore, the heterogeneity in the timing of surrogate endpoints used in the included trials also poses statistical challenges. While the definitions of tumor response assessments and adverse events were generally similar among the original papers, the variability in timing could affect the results and introduce additional uncertainty. Lastly, it is important to approach the interpretation of the study results cautiously. Using ORR and irAEs as surrogate measures for predicting survival may not provide absolute predictive information due to the different measurement scales and statistical properties of these variables. The relationship between ORR, irAEs, and survival might not be a simple linear one and could have a more complex form. Considering these aforementioned limitations, the findings of this meta-analysis should be interpreted with caution. Further research with more comprehensive data and adjusted analyses is necessary to confirm and expand upon these findings.

In summary, while the near-term effect may provide some clues to the antitumor response after treatment, further long-term studies and observations are needed to determine the survival effect of immune checkpoint inhibitors in the treatment of advanced cancer. OS remains a promising end point, and our study's finding of a high correlation between early and late OS data reinforces the value of including mid-stage analyses in phase III trials to capture early signals of efficacy and to include ineffectiveness boundaries for early termination.

## Conclusion

This study provides important insights into the therapeutic effect of ICIs in patients with uHCC and the correlation between surrogate endpoints and overall survival (OS). Our findings indicate that PFS is a good surrogate endpoint for OS in phase III clinical trials. Additionally, CR demonstrates a strong correlation with clinical survival benefit. However, it is essential to note that other commonly used early surrogate markers, such as the OR, are not reliable substitutes for predicting clinical survival benefit. These findings emphasize the need to carefully consider and select appropriate surrogate endpoints in uHCC clinical trials, particularly PFS and CR, to ensure accurate evaluation of treatment efficacy and inform decision-making for improved patient outcomes.

### Supplementary Information

Supplementary Information.

## Data Availability

The data that supports the findings of this study are available from the corresponding author upon reasonable request.
