# Supplementary material for: Correlation between surrogate endpoints and overall survival in unresectable hepatocellular carcinoma patients treated with immune checkpoint inhibitors: a systematic review and meta-analysis
Source: Sci Rep. 2024 Feb 21;14:4327. doi: 10.1038/s41598-024-54945-6 (PMC10881995; doi:10.1038/s41598-024-54945-6)

**eTable 1** Search Strategy

**eFigure 1** Flowchart of data analysis

**eTable 2** List of excluded studies

**eFigure 2** Risk of bias assessment of the included clinical trials

**eTable 1 Search Strategy**

| Embase | |
| --- | --- |
| 1 | unresectable hepatocellular carcinoma.mp. |
| 2 | (unresectable hepatocellular carcinoma or advanced hepatocellular carcinoma or metastatic hepatocellular carcinoma).ab,ti. |
| 3 | immunotherapy.mp. or exp cancer immunotherapy/ or exp immunotherapy/ or immune checkpoint inhibitor.mp. or exp immune checkpoint inhibitor/ |
| 4 | (immune checkpoint inhibitor or ICIs or programmed cell death 1 receptor antagonists or programmed cell death 1 receptor or programmed cell death 1 receptor inhibitor or pd-1 antibody or pd-l1 antibody or ctla-4 antibody or pd-1 inhibitor or pd-l1 inhibitor or ctla-4 inhibitor).ab,ti. |
| 5 | (atezolizumab or tecentriq or rg7446 or mpdl3280a or Ipilimumab or durvalumab or tremelimumab or tislelizumab or toripalimab or penpulimab or cemiplimab or pembrolizumab or mk-3475 or lambrolizumab or keytruda or nivolumab or opdivo or bms-936558 or mdx-1106 or camrelizumab or shr-1210 or airuika or hr-301210).ab,ti. |
| 6 | 3 or 4 or 5 |
| 7 | clinical trials.mp. or exp "clinical trial (topic)"/ |
| 8 | 1 or 2 |
| 9 | 6 and 7 and 8 |
| 10 | limit 9 to human |
| Ovid MEDLINE | |
| 1 | unresectable hepatocellular carcinoma.mp. |
| 2 | (unresectable hepatocellular carcinoma or advanced hepatocellular carcinoma or metastatic hepatocellular carcinoma).ab,ti. |
| 3 | 1 or 2 |
| 4 | immunotherapy.mp. or Immunotherapy/ or immune checkpoint inhibitor.mp. or exp Immune Checkpoint Inhibitors/ |
| 5 | (immune checkpoint inhibitor or ICIs or programmed cell death 1 receptor antagonists or programmed cell death 1 receptor or programmed cell death 1 receptor inhibitor or pd-1 antibody or pd-l1 antibody or ctla-4 antibody or pd-1 inhibitor or pd-l1 inhibitor or ctla-4 inhibitor).ab,ti. |
| 6 | (atezolizumab or tecentriq or rg7446 or mpdl3280a or Ipilimumab or durvalumab or tremelimumab or tislelizumab or toripalimab or penpulimab or cemiplimab or pembrolizumab or mk-3475 or lambrolizumab or keytruda or nivolumab or opdivo or bms-936558 or mdx-1106 or camrelizumab or shr-1210 or airuika or hr-301210).ab,ti. |
| 7 | 4 or 5 or 6 |
| 8 | clinical trials.mp. or exp Clinical Trial/ |
| 9 | 3 and 7 and 8 |
| 10 | limit 9 to humans |
| Cochrane Central Register of Controlled Trials (Ovid) | |
| 1 | unresectable hepatocellular carcinoma.mp. |
| 2 | (unresectable hepatocellular carcinoma or advanced hepatocellular carcinoma or metastatic hepatocellular carcinoma).ab,ti. |
| 3 | 1 or 2 |
| 4 | immunotherapy.mp. or exp Immunotherapy/ or immune checkpoint inhibitor.mp. or exp Immune Checkpoint Inhibitors/ |
| 5 | (immune checkpoint inhibitor or ICIs or programmed cell death 1 receptor antagonists or programmed cell death 1 receptor or programmed cell death 1 receptor inhibitor or pd-1 antibody or pd-l1 antibody or ctla-4 antibody or pd-1 inhibitor or pd-l1 inhibitor or ctla-4 inhibitor).ab,ti. |
| 6 | (atezolizumab or tecentriq or rg7446 or mpdl3280a or Ipilimumab or durvalumab or tremelimumab or tislelizumab or toripalimab or penpulimab or cemiplimab or pembrolizumab or mk-3475 or lambrolizumab or keytruda or nivolumab or opdivo or bms-936558 or mdx-1106 or camrelizumab or shr-1210 or airuika or hr-301210).ab,ti. |
| 7 | 4 or 5 or 6 |
| 8 | clinical trials.mp. or exp Clinical Trial/ |
| 9 | 3 and 7 and 8 |

**eFigure 1** Flowchart of data analysis

**Narrative description**:

Categorical variables, report frequency and percentage;

Continuous variables, report median and interquartile range

**Extraction data：**

The hazard ratio and its corresponding 95% confidence interval (CI). In the absence of explicitly provided HRs, HRs were estimated based on a given median survival time or survival at that time point

Random effect multivariate meta-analysis was used to evaluate surrogacy between the HRs for OS and each endpoint.

**Frequentist hybrid model:**

Input: Collect effect sizes and within-study variances from individual studies included in the meta-analysis.

Initialization: Start by assuming an initial value for the between-study variance and correlation parameters.

Estimation: Use iterative numerical methods, such as the Expectation-Maximization (EM) algorithm or restricted maximum likelihood (REML), to estimate the between-study variance and correlation parameters.

Convergence: Check for convergence of the estimated parameters. If convergence is not achieved, go back to step 3 and repeat the estimation until convergence is reached.

Final Estimates and Inference: Obtain the final estimates of the overall effect sizes, between-study variances, marginal correlation matrix, and covariance matrix based on the converged parameter values.

**Bayesian hybrid model:**

Prior Specification: Define prior distributions for the unknown parameters, including the between-study variances, correlation parameters, and any other relevant hyperparameters.

Posterior Calculation: Apply Bayesian inference techniques, such as MCMC sampling or variational inference, to compute the posterior distributions of the parameters given the observed data and the prior information.

Sampling: Generate multiple samples from the joint posterior distribution of the parameters using MCMC or other sampling methods. These samples can be obtained from the full conditional distributions of the parameters, which are often easier to sample from than the joint posterior distribution.

Posterior Summaries: Based on the posterior samples, calculate various posterior summaries, such as the posterior means, medians, credible intervals, and posterior predictive distributions.

**eTable 2 List of excluded or included studies**

| Title | Journal | Reasons for exclusion or inclusion |
| --- | --- | --- |
| Anti-PD-1 Antibody SHR-1210 Combined with Apatinib for Advanced Hepatocellular Carcinoma, Gastric, or Esophagogastric Junction Cancer: An Open-label, Dose Escalation and Expansion Study | Clinical Cancer Research 2019;25(2):515-23 | Non-comparative studies |
| Atezolizumab plus Bevacizumab in Unresectable Hepatocellular Carcinoma | New England Journal of Medicine 2020;382(20):1894-905 | Inclusion |
| Atezolizumab with or without bevacizumab in unresectable hepatocellular carcinoma (GO30140): an open-label, multicentre, phase 1b study | Lancet Oncology 2020;21(6):808-20 | Inclusion |
| Bevacizumab and atezolizumab as first-line therapy for advanced hepatocellular carcinoma: A Taiwanese subgroup analysis on efficacy and safety | Journal of the Formosan Medical Association 2022;121(12):2430-37 | Post hoc |
| Cabozantinib in combination with atezolizumab versus sorafenib in treatment-naive advanced hepatocellular carcinoma: COSMIC-312 Phase III study design | Future Oncology 2020;16(21):1525-36 | Non-comparative studies |
| Cabozantinib plus atezolizumab versus sorafenib for advanced hepatocellular carcinoma (COSMIC-312): a multicentre, open-label, randomised, phase 3 trial | Lancet Oncology 2022;23(8):995-1008 | Inclusion |
| Camrelizumab in patients with previously treated advanced hepatocellular carcinoma: a multicentre, open-label, parallel-group, randomised, phase 2 trial | Lancet Oncology 2020;21(4):571-80 | Inclusion |
| Characterization of response to atezolizumab + bevacizumab versus sorafenib for hepatocellular carcinoma: Results from the IMbrave150 trial | Cancer Medicine 2021;10(16):5437-47 | Inclusion |
| CheckMate 040 cohort 5: A phase I/II study of nivolumab in patients with advanced hepatocellular carcinoma and Child-Pugh B cirrhosis | Journal of Hepatology 2021;75(3):600-09 | Non-comparative studies |
| Chimeric Antigen Receptor-Glypican-3 T-Cell Therapy for Advanced Hepatocellular Carcinoma: Results of Phase I Trials | Clinical Cancer Research. 2020;26(15):3979-89. | Non-comparative studies |
| Efficacy and Safety of Nivolumab Plus Ipilimumab in Patients With Advanced Hepatocellular Carcinoma Previously Treated With Sorafenib: The CheckMate 040 Randomized Clinical Trial | JAMA Oncology 2020;6(11):e204564 | Inclusion |
| Final Results of TACTICS: A Randomized, Prospective Trial Comparing Transarterial Chemoembolization Plus Sorafenib to Transarterial Chemoembolization Alone in Patients with Unresectable Hepatocellular Carcinoma | Liver Cancer 2022;11(4):354-67 | Non ICI intervention |
| Health-related quality-of-life impact of pembrolizumab versus best supportive care in previously systemically treated patients with advanced hepatocellular carcinoma: KEYNOTE-240 | Cancer 2021;127(6):865-74 | Lacking OS and/or other endpoints |
| Ipilimumab with atezolizumab-bevacizumab in patients with advanced hepatocellular carcinoma: The PRODIGE 81-FFCD 2101-TRIPLET-HCC trial | Digestive & Liver Disease 2023;55(4):464-70 | Lacking OS and/or other endpoints |
| Nivolumab (Nivo) in patients (Pts) with advanced hepatocellular carcinoma (HCC): the CheckMate 040 study | Hepatology (Baltimore, Md) 2016;Vol | Non-comparative studies |
| Nivolumab in advanced hepatocellular carcinoma: Sorafenib-experienced Asian cohort analysis | Journal of Hepatology 2019;71(3):543-52 | Inclusion |
| Nivolumab in patients with advanced hepatocellular carcinoma (CheckMate 040): an open-label, non-comparative, phase 1/2 dose escalation and expansion trial | Lancet 2017;389(10088):2492-502 | Non-comparative studies |
| Nivolumab versus sorafenib in advanced hepatocellular carcinoma (CheckMate 459): a randomised, multicentre, open-label, phase 3 trial | Lancet Oncology 2022;23(1):77-90 | Inclusion |
| Patient-reported outcomes with atezolizumab plus bevacizumab versus sorafenib in patients with unresectable hepatocellular carcinoma (IMbrave150): an open-label, randomised, phase 3 trial | Lancet Oncology 2021;22(7):991-1001 | Inclusion |
| Pembrolizumab as second-line therapy for advanced hepatocellular carcinoma: A subgroup analysis of asian patients in the phase 3 KEYNOTE-240 trial | Liver Cancer 2021;10(3):275-84 | Inclusion |
| Pembrolizumab As Second-Line Therapy in Patients With Advanced Hepatocellular Carcinoma in KEYNOTE-240: A Randomized, Double-Blind, Phase III Trial | Journal of Clinical Oncology 2020;38(3):193-202 | Inclusion |
| Pembrolizumab in patients with advanced hepatocellular carcinoma previously treated with sorafenib (KEYNOTE-224): a non-randomised, open-label phase 2 trial | Lancet Oncology 2018;19(7):940-52 | Non-comparative studies |
| Pembrolizumab Versus Placebo as Second-Line Therapy in Patients From Asia With Advanced Hepatocellular Carcinoma: A Randomized, Double-Blind, Phase III Trial | Journal of Clinical Oncology 2023;41(7):1434-43 | Inclusion |
| Perioperative nivolumab monotherapy versus nivolumab plus ipilimumab in resectable hepatocellular carcinoma: a randomised, open-label, phase 2 trial | The Lancet Gastroenterology & Hepatology 2022;7(3):208-18 | Lacking OS and/or other endpoints |
| RATIONALE 301 study: tislelizumab versus sorafenib as first-line treatment for unresectable hepatocellular carcinoma | Future Oncology 2019;15(16):1811-22 | Non-comparative studies |
| Safety and preliminary efficacy of nivolumab in patients with advanced hepatocellular carcinoma: interim analysis of the phase 1/2 CheckMate 040 study | Hepatology international 2017;Vol | Post hoc |
| Sintilimab plus a bevacizumab biosimilar (IBI305) versus sorafenib in unresectable hepatocellular carcinoma (ORIENT-32): a randomised, open-label, phase 2-3 study | Lancet Oncology 2021;22(7):977-90 | Inclusion |
| Transarterial Radioembolization Versus Atezolizumab-Bevacizumab in Unresectable Hepatocellular Carcinoma: A Matching-Adjusted Indirect Comparison of Time to Deterioration in Quality of Life | Advances in Therapy 2022;39(5):2035-51 | Non-clinical trials |
| Updated efficacy and safety data from IMbrave150: Atezolizumab plus bevacizumab vs sorafenib for unresectable hepatocellular carcinoma | J Hepatol. 2022;76(4):862-73 | Inclusion |
| Updated efficacy and safety of KEYNOTE-224: a phase II study of pembrolizumab in patients with advanced hepatocellular carcinoma previously treated with sorafenib | European Journal of Cancer 2022;167:1-12 | Non-comparative studies |

During the initial search, several studies were identified that initially appeared to meet our inclusion criteria. However, these studies were ultimately excluded based on our pre-defined exclusion criteria, which aimed to ensure that only studies with relevant data were included in the final analysis.

**eFigure 2** Risk of bias assessment of the included clinical trials


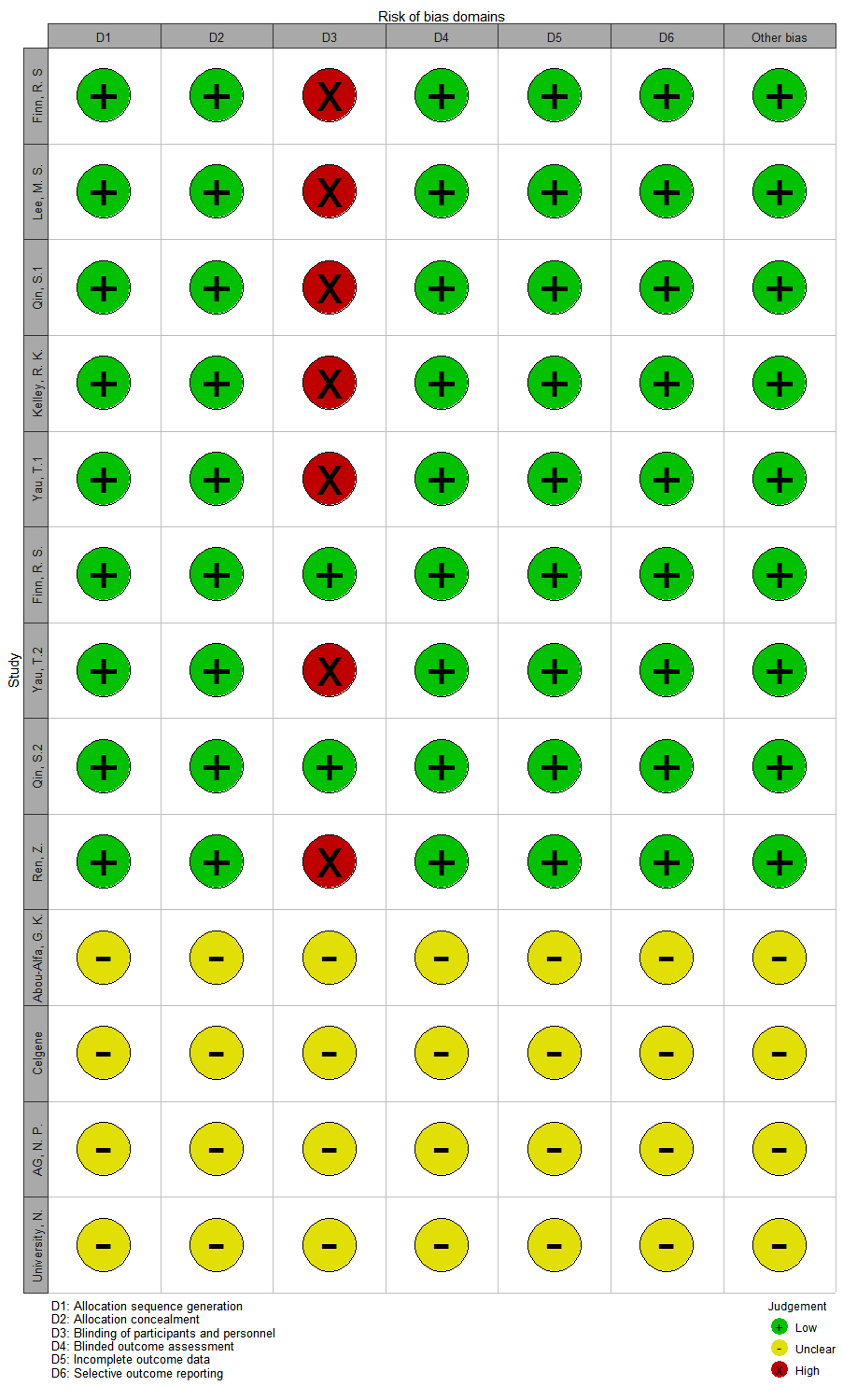

Supplement: Supplementary file 1 — Supplementary Information. [file 41598_2024_54945_MOESM1_ESM.docx]
